# Physical exercise protects against *Toxoplasma gondii* infection-induced muscle atrophy and microvascular rarefaction

**DOI:** 10.1038/s42003-026-09810-9

**Published:** 2026-03-10

**Authors:** Paloma de Carvalho Vieira, Carolina Epifânio, Samuel Iwao Horita, Karine Lino Rodrigues, Evelyn Pereira, Anissa Daliry, Leonardo Leal de Castro, Barbara Gomes da Rosa, Seth Kahn, Cynthia Machado Cascabulho, Mariana G. Chauvet, Mychael V. Lourenco, Helene Santos Barbosa, Daniel Adesse

**Affiliations:** 1 Laboratório de Biologia Estrutural, Instituto Oswaldo Cruz, Fiocruz, Rio de Janeiro, Brazil; 2 Laboratório de Inovações em Terapias, Ensino e Bioprodutos, Instituto Oswaldo Cruz, Fiocruz, Rio de Janeiro, Brazil; 3 Laboratório de Pesquisas sobre o Timo, Instituto Oswaldo Cruz, Fiocruz, Rio de Janeiro, Brazil; 4 Laboratório de Fisiopatologia Clínica e Experimental, Instituto Oswaldo Cruz, Fiocruz, Rio de Janeiro, Brazil; 5 Instituto de Bioquímica Médica Leopoldo de Meis, Universidade Federal do Rio de Janeiro, Rio de Janeiro, Brazil; 6 Laboratory of Ocular Immunology and Transplantation, Department of Ophthalmology, Bascom Palmer Eye Institute, University of Miami Leonard Miller School of Medicine, Miami, FL USA

**Keywords:** Parasite host response, Parasitic infection, Infection, Neuro-vascular interactions, Blood flow

## Abstract

Toxoplasmosis is a leading cause of death from foodborne illness. Its causative agent, *Toxoplasma gondii*, forms cysts in skeletal muscle, contributing to parasite persistence and transmission. Cases of myositis have been reported in *T. gondii*-infected immunocompetent individuals, and impaired myogenesis has been demonstrated in mice. Herein we investigated the effects of *T. gondii* infection on glycolytic tibialis anterior (TA) and oxidative soleus (SOL) muscles in mice, and whether prior physical exercise could prevent infection-induced myopathology. Functional, metabolic, histological, inflammatory and microvascular parameters were analyzed 10 and 40 days post infection (dpi). *T. gondii* caused greater disruption to TA than to SOL, with increased atrophy and pathology. Expression of muscle atrophy-related genes (Atrogenes), including MURF1 and Atrogin-1, was strongly induced at 10 and normalized by 40 dpi. A fiber-type shift was observed in SOL at 40 dpi. Infected sedentary mice showed reduced grip strength and VO₂, while these effects were prevented by exercise. Exercised-infected mice had lower expression of Murf1, IL-6ra, STAT3, and pro/anti-inflammatory cytokine ratios compared to sedentary-infected controls. Exercise also preserved muscle and brain microvascular flow and prevented leukocyte-endothelium interactions. In conclusion, *T. gondii* differentially affects glycolytic and oxidative muscles; exercise protects muscle and brain against parasite-related damage.

## Abstract (web summary)

*T. gondii* infection leads to differential injury in distinct metabolic profile muscles. Physical endurance exercise protects both muscle and brain against *T. gondii*-induced damage.

## Introduction

Toxoplasmosis is a widespread food-borne parasitic disease, with seroprevalence rates between 16 and 62% across different continents^1,2^. While immunocompetent individuals present mild symptoms, myositis and polymyositis cases may occur, mistakenly diagnosed as idiopathic conditions, with diffused myalgia, electromyographic abnormalities, reduced grip strength, and reflexes^3,4^. *Toxoplasma gondii*, the etiological agent of Toxoplasmosis, forms bradyzoite cysts, its latency stage, in skeletal muscle^5–8^ and neurons^9^. Host cell metabolism and cell cycle status are determinants of whether the parasite will follow a lytic cycle or cyst formation^10–12^. Skeletal muscle (SkM) is an important route for *T. gondii* transmission, as the ingestion of undercooked or raw meat containing cysts is one of the main routes of transmission^13–16^.

The striated SkM represents about 40% of the human body mass and is primarily responsible for locomotion, but also for speech and social interactions^17^. SkM can also be described as an endocrine tissue, as hundreds of cytokines and other molecules are released by the tissue, called myokines^18^. More recently, exerkines have been proposed as a term to describe the molecules released in response to exercise^19^. Physical exercise has been studied in recent decades as a preventive or therapeutic approach for several diseases. Exercise has been shown to be protective against conditions such as sarcopenia, diabetes, heart disease, and some types of cancer^20–24^. Furthermore, the beneficial actions of physical exercise have been reported in animal models of Alzheimer’s disease and other neurodegenerative diseases, such as Parkinson’s, Huntington’s, and amyotrophic lateral sclerosis^25–31^. Mechanistically, exercise improves neurogenesis, synaptic plasticity, and angiogenesis^32^, thus contributing to learning and memory.

Congenital toxoplasmosis impairs myogenesis in mice^33^, and the myogenic program is disrupted by *T. gondii* infection in vitro^34,35^. Acquired infection leads to cachexia, with progressive weight loss, muscle atrophy, and fibrosis^36^. However, the underlying mechanisms of Toxoplasma-induced myopathology have not been studied to date. Herein, we further investigate the pathways affected by acquired toxoplasmosis in mice, comparing two different types of muscle: the glycolytic tibialis anterior (TA) and the oxidative soleus (SOL). We demonstrate that oxidative and glycolytic muscles respond differently to *T. gondii* infection with a higher compromise of glycolytic fibers. Moreover, we analyzed the role of exercise in the prevention of *T. gondii*-induced pathology. Endurance training prevented systemic inflammation and expression of muscle atrophy-related genes in infected mice. Strikingly, both muscle and cerebral blood flow, which are reduced in sedentary-exercised mice, are preserved in exercised-infected ones. Our findings bring an important contribution to the understanding of toxoplasmosis myopathogenesis and highlight a previously unknown protective effect of physical exercise on neurovascular alterations in infected mice.

## Methods

### Animals

Initially, *T. gondii* infection was performed in female 40-day-old Swiss Webster (SW) mice, *Mus musculus*. For physical exercise experiments, males were used in order to avoid the hormone-induced variations in mouse performance and possible interference from this variable in exercise effects against infection. Animals were housed with food and water *ad libitum* until *T. gondii* infection, in a 12:12 light/dark cycle, with environmental enrichment, which was changed every 2 weeks. To the first set of experiments, after a week of habituation, female mice (20–35 g) were intragastrically infected with *T. gondii* cysts in 100 μL PBS (phosphate-buffered saline) by oral gavage, as the controls received the same volume of PBS. To the second set of experiments, 40-day-old male SW animals (30–40 g) were infected in the end of the physical training protocol. Animals were weighted at the infection day, and then weekly, as total food per cage, to calculate group intake. At the desired days post infection (dpi), mice were euthanized, and the tissues of interest were collected. The physical training and functional tests were performed in the same period of the day (morning), and by the same investigator. Animal experimentation was approved by the Ethics Committee in Animal Welfare at the Oswaldo Cruz Institute (CEUA-IOC), under license number L040/2020. We have complied with all relevant ethical regulations for animal use.

### Parasite isolation

Cysts were obtained from the brains of previously infected C57BL/6 mice. Mice were euthanized after ~6 weeks of infection and their brains dissected, homogenized with a syringe and needles of different calibers in PBS at 4 °C. A solution containing 10 Me49 strain cysts in 100 μL was prepared for each mouse. Bradyzoites purification for RNA extraction was performed as previously described^37^.

### Functional tests

Kondziela’s inverted screen test and weightlifting were performed as previously published^38^. In the grip strength test, the animals were allowed to hold the metal bar in the grip device (Bonther, São Paulo, Brazil), and gently had their tails pulled. The grip strength applied by them onto the metal bar to stay in the device, and the time was registered. The animals were subjected to the test for six consecutive trials for 2 s each.

### Aerobic physical exercise protocol

Maximum exercise capacity was determined by VO_2max_ measured by an open-circuit calorimetry system, during a maximal incremental test on a treadmill ergometer coupled to a metabolic chamber (AVS projects, São Paulo, SP), with an air flow maintained at 3500 ml/min. The O_2_ fraction was continuously measured at 20 Hz (AQCAD, AVS projects, São Paulo, SP), and VO_2max_ was calculated by the equation:

VO_2max_ (ml/Kg*min) = ((20.93-% O_2_ consumed) × Air flow/100)/Animal weight (Kg). Mice were kept on the treadmill for 5 min without exercise to stabilize the equipment. The test started at 10 m/min, increasing by 3 m/min every 3 min until exhaustion, defined as the inability to maintain the pace for 5 s, even with electrical stimulation. All animals were adapted to running 10 min sessions before the test, while the sedentary mice remained on a stationary treadmill for a similar time. Physical exercise protocol consisted in allowing mice to run in the treadmill with 60% of their maximum speed capacity for 30 min, with training sessions 5 days/week, with 2 days for resting. Mice were kept under this exercise protocol for 8 weeks. VO_2max_ was retested and adjusted after 4 weeks of exercise. Both exercised and sedentary groups were either infected with 10 cysts of *T. gondii* (Me49 strain) or inoculated with PBS by oral gavage in a final volume of 100 µL. Only male mice were employed in this protocol.

### Surgical procedures for real-time microcirculation analysis

Microcirculation was examined by intravital microscopy to visualize leukocyte recruitment and by laser speckle to measure basal microvascular flow and vascular reactivity^39^. At the end of the experimental protocol, animals that had fasted overnight for 8 h were anesthetized via intraperitoneal injection of ketamine hydrochloride (100 mg/kg) and xylazine (10 mg/kg) diluted in saline solution, and then fixed in a stereotaxic frame. To visualize the cerebral microcirculation in vivo, a midline incision was made to expose the cranial window^40^. For in vivo TA analysis, the leg was fixed above the ankle and placed perpendicularly to the other. A small incision was made in the skin and in the outer connective tissue to expose the muscle tissue^41^.

### Intravital microscopy

For TA intravital microscopy analyses, a 10× ocular and 10× objective lenses (Olympus BX150WI; Center Valley, PA, USA) were used. Images were projected on a screen for quantitative assessments. Leukocyte-endothelial interaction was evaluated after intravenous administration of 0.3 mg/kg rhodamine 6 G (Sigma Chemical Co., St. Louis, MO, USA). Rolling or adhering leukocytes on the brain or muscle venule surface were counted over a period of 30 s^42^.

### Laser speckle contrast imaging (LSCI)

Laser Speckle Contrast Imaging (LSCI, Pericam PSI system, Perimed, Sweden) was employed to visualize brain and TA microvascular blood flow in real time^43^. Mice were kept on a stable surface, in a room set at 25 °C. LSCI generates real-time color heatmaps of tissue blood flow by detecting the interference patterns of coherent laser light scattered by red blood cells within the region of interest (ROI) from a 10-cm distance between the scanner and the tissue surface. Following a 5-min stabilization of microcirculatory flow, Acetylcholine was directly applied to the ROI to evaluate endothelial response in a dose-dependent manner. Relative blood flow was expressed in Arbitrary Perfusion Units (APUs).

### Histological and morphometric analysis

TA and SOL were collected as previously described^44^ and sectioned into 7 μm-thick slices on a cryostat (−20 °C). The slices were stained with hematoxylin and eosin and imaged with a Zeiss AxioVision bright-field microscope. Fibers Cross Sectional Area (CSA) was determined with ImageJ/ FIJI^45^, using the threshold tool to delimit the stained fiber. Inflammation and necrotic areas were manually selected, as described^46^. To assess the fiber type, sections were incubated with anti-MyHC type I, type IIa, and type IIb, as described before (Table 1^47^); TA and SOL sections were visualized on a fluorescence microscope (Axio Imager.A2, Zeiss), and 10 images were obtained from each sample using a 20× objective lens. Fiber types were quantified, and the percentage relative to the total fiber count was represented.

**Table 1:** List of primary antibodies used in this study

Antibody	Host species	Company	Reference number	Dilution
MyHC Type I	Mouse	DSHB	BA-D5	1:10
MyHC Type IIa	Mouse	DSHB	SC-71	1:10
MyHC Type IIb	Mouse	DSHB	BF-F3	1:10
STAT3	Mouse	Cell signaling	9139	1:1000
pSTAT3	Rabbit	Cell signaling	9145	1:1000
GAPDH	Mouse	Biosynth	10R-G109A	1:15000

### RNA extraction and RT-qPCR

TA, SOL, and brain cortex samples had their RNA extracted with Trizol reagent (ThermoFisher). 500 ng of RNA was treated with DNase I, and the cDNA was synthesized with SuperScript III or High Fidelity kits (ThermoFisher). RT-qPCR gene expression assessment was performed with the SYBR Green detection system in StepOne Plus or with QuantStudio V (ThermoFisher) cyclers. Primer sequences for MuRF1, Atrogin-1, Myomaker, Pax7, Opa1, Mitofusin-1, FIS1, Drp1, MFF, PGC-1α, FNDC5, IL-6 receptor, STAT3, and MyHC-1, -2, -3, -4, -7, are available at Table 2. PPIA was used as a housekeeping gene, as previously described^48^.

**Table 2:** Sequences and references of the primers used in this study

Target	Gene Symbol	Forward sequence (5′–3′)	Reverse sequence (5′–3′)	Reference
Cyclophilin A	*Ppia*	GGCCGATGACGAGCCC	TGTCTTTGGAACTTTGTCTGCAA	^83^
Murf1	*Trim63*	TGCTTGGCACTTGAGAGGAA	AGAAGCTGGGCTTCATCGAG	^84^
Atrogin1	*Fbxo32*	TGGGTGTATCGGATGGAGAC	TCAGCCTCTGCATGATGTTC	^84^
Pax7	*Pax7*	GACTCGGCTTCCTCCATCTC	AGTAGGCTTGTCCCGTTTCC	^49^
Myomaker	*Mymk*	ATCGCTACCAAGAGGCGTT	CACAGCACAGACAAACCAGG	^54^
MyHC type I	*Myh7*	CGCAATGCAGAGTCAGTGAA	TTGCGGAACTTGGACAGGTT	^85^
MyHC type IIb	*Myh4*	GACGACCTTGAGCTGACACT	TTGACTTTGTCCTCCTCTGC	-
MyHC type IIa	*Myh2*	GCTGAAGCAGAGGCAAGTAG	TTCATTGGGGATGATACACC	-
MyHC type IIx	*Myh1*	GGGCTCCTAGAGGAGATGAG	ACTCTCGCCAAGTACCCTCT	-
Embryonic MyHC	*Myh3*	ATGAGTAGCGACACCGAGATG	AAAGCAGTAGGTTTTGGCAT	^86^
Opa1	*Opa1*	ACTGCAGGTCCCAAATTGGTT	GCGCTCCAAGATCCTCTGAT	
Mfn1	*Mfn1*	CCAGGTACAGATGTCACCACAG	TTGGAGAGCCGCTCATTCACCT	^87^
Fis1	*Fis1*	GAGAACATCCTCGGGTGCAG	CTTTGGGCAACAGCTCCTCC	
Drp1	*Drp1*	GCGAACCTTAGAATCTGTGGACC	CAGGCACAAATAAAGCAGGACGG	^87^
MFF	*MFF*	GTCCCAGAGAGGATCGTGGT	TGCTCGGCTCTCTTCGCTTT	
Atg5	*Atg5*	ACCCCTGAAATGAGTTTTCCAGA	TGATGGCCCAAAACTGGTCA	^88^
PGC-1α	*Ppargc1a*	GAATCAAGCCACTACAGACACCG	CATCCCTCTTGAGCCTTTCGTG	^89^
FNDC5	*Fndc5*	GGACTCTTGGAAAACACCACTG	TCGTGTCCACACAGATGATCTCACCAC	^27^
IL-6ra	*Il6ra*	TGAATGATGACCCCAGGCAC	ACACCCATCCGCTCTCTACT	-
STAT3	*Stat3*	ATCTTTGGGCAATCTGGGCA	CTTCTGCTCTCAGCCCCATC	-
Enolase 2	*Eno2*	CCGTTACTCAACTTCCAACA	CCATCGGTCAACAAGTCAA	^90^
BAG1	*Bag1*	GGGATGTACCAAGCATCCTG	AGGGTAGTACGCCAGAGCAA	^90^

### Western blotting

Tissues were disrupted in RIPA buffer with Na_3_VO_4_, PMSF, and protease inhibitors (Santa Cruz Biotechnology) with TissueLyser II (Qiagen); protein concentration was determined with the BCA kit (ThermoFisher). 30 µg of protein was loaded onto 10% bis-acrylamide gels and separated by electrophoresis (100 V for 120 min). Proteins were transferred to nitrocellulose membranes, and blocked with TBST (25 mM Tris, 150 mM NaCl, 0,05% Tween) + 5% bovine serum albumin (BSA, Sigma Chemical Co., St. Louis, MO, USA), followed by incubations with primary antibodies in blocking solution overnight at 4 °C under agitation. Antibodies dilutions can be found in Table 1. Membranes were scanned using a ChemiDoc MP Imaging System (Bio-Rad) and quantified with ImageJ software.

### ELISA

FNDC5/irisin detection was performed by a sandwich ELISA reaction using DuoSet® ELISA Development Systems kit (R&D Systems), according to manufacturer’s instructions. Blood samples were collected at 10 or 40 dpi, allowed to sit for at least 1 h, and then centrifuged for 10 min at 224 *g* in a refrigerated centrifuge (4 °C) to isolate the serum. Samples were stored at −80 °C until irisin detection.

### Cytometric bead array

Serum samples were assayed for TNF-α, INF-γ, IL-2, IL-4, IL-6, IL-10, and IL-17a using a Cytometric Bead Array (CBA) Th1/ Th2/ Th17 kit (BD Biosciences), according to the manufacturer’s instructions. Data were acquired using a Cytoflex S (Beckman Coulter) flow cytometer. After data acquisition, dedicated software (FCAP Array, BD Biosciences) was used to analyze the results by gating bead populations, calculating MFI values, generating standard curves, and determining analyte concentrations. These analyses were performed at the Flow Cytometry Facility of the Instituto Oswaldo Cruz (Fiocruz).

### Parasite burden quantification

*T. gondii* infection was quantified using RT-qPCR with *bag1* and *enolase2* primers to detect bradyzoite and tachyzoite forms, respectively, according to^49^. Ct values were compared to a standard curve amplification, derived from known *T. gondii* RNA concentrations. The standard curve was constructed with six 10-fold dilutions, starting with 6.0 × 10^6^ parasites for either bradyzoites or tachyzoites. Primer sequences are available in Table 2.

### Statistics and reproducibility

Data was analyzed by GraphPad Prism Software v.10.0.0, using a two-way ANOVA test. Post-hoc pairwise comparisons were conducted using Bonferroni’s test to account for multiple comparisons and the Mann-Whitney test for two groups comparison. Statistical significance was considered using a 95% confidence interval (*p-*value  < 0.05). Outliers were identified using the ROUT method (*Q* = 1%) with GraphPad Prism v.10.0.0. Identified outliers were excluded from further analysis. The results were presented as means ± SEM.

### Reporting summary

Further information on research design is available in the Nature Portfolio Reporting Summary linked to this article.

## Results

### *T. gondii* infection leads to subtle SkM functional loss and transient atrophy response

**Fig. 1:**
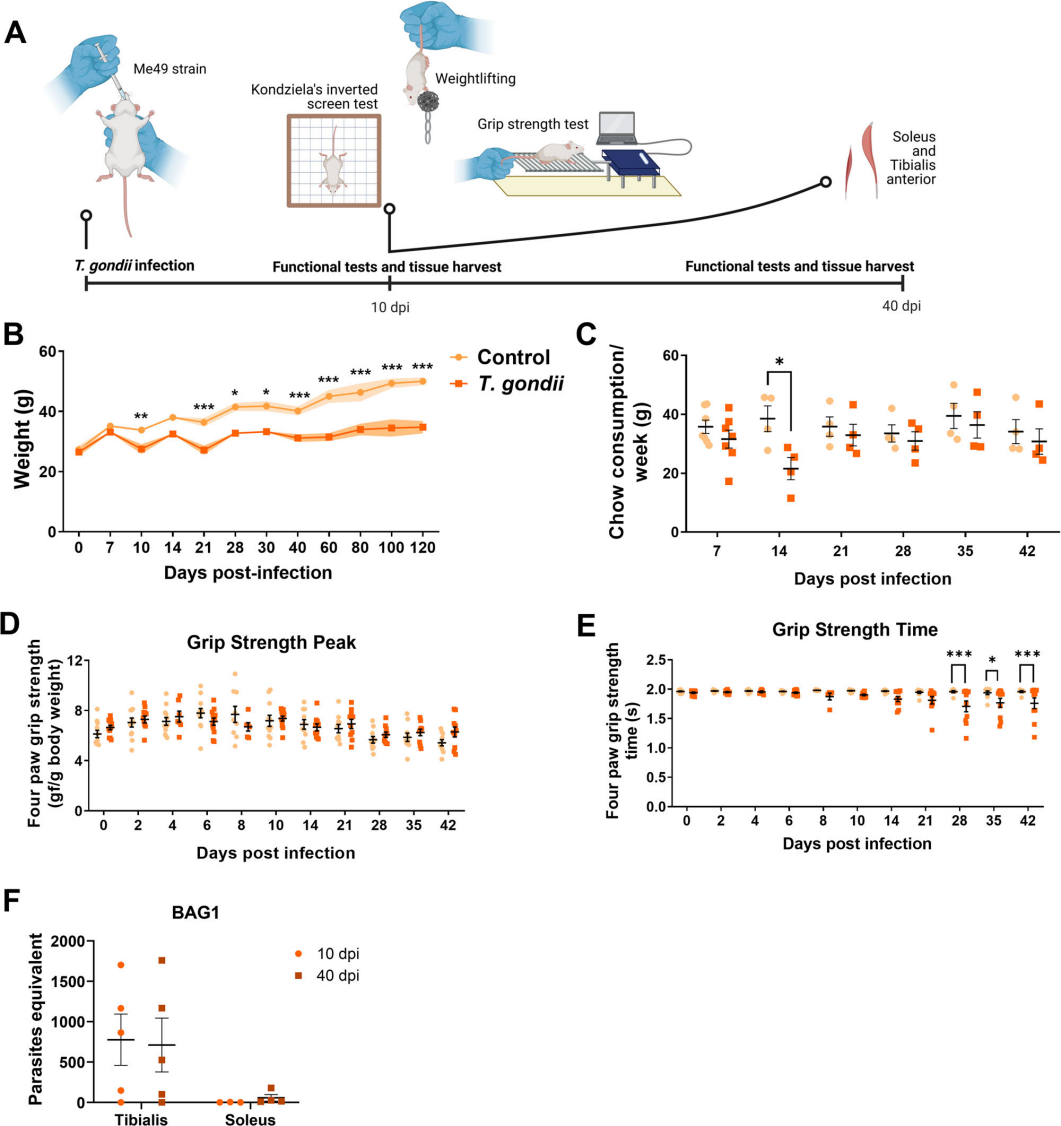
*T. gondii*effects in body weight and muscle function. **A** Experimental design. Created in BioRender. Adesse, D. (2025) https://BioRender.com/zkv4axo. **B** Mice body weight decreased after 10 dpi. **C** Weekly chow consumption for animal. **D** Grip strength measure of mice (four paws test) did not showed differences in strength peak, but in time of applying force (**E**). **F** Bradyzoite’s marker BAG1 expression is higher in TA than in SOL in both time points. *: *p* < 0.05, **: *p* < 0.01, ***: *p* < 0.001. Two-Way ANOVA, Bonferroni’s post-test. Each dot in the graphs corresponds to independent mice.

SW female mice were infected, and muscle function was assessed as depicted in Fig. 1A. The infection reduced body weight starting at 10 dpi (Fig. 1B) and food consumption at the second week of infection (Fig. 1C). TA and SOL weight were not altered by the infection (Fig. S1E, F).

Muscle function was evaluated by Kondziela’s inverted screen test, muscle weightlifting test, and grip strength measure. The first two tests applied did not show differences between infected and control animals (Fig. S1A, B) in any period. However, infected animals showed less endurance in the grip strength test. From 28 dpi until the last time point evaluated, the strength peak was unaltered (Fig. 1D), but the permanence in the grip time was decreased by infection (Fig. 1E). Tachyzoite signal, accessed by *eno2* expression, was not detected in either muscle types (*data not shown*), but bradyzoite *bag1* transcripts were found in TA (approximately 700 parasite-equivalents) at both times analyzed, and in SOL (approximately 50 parasite-equivalents) at 40 dpi (Fig. 1F).

**Fig. 2:**
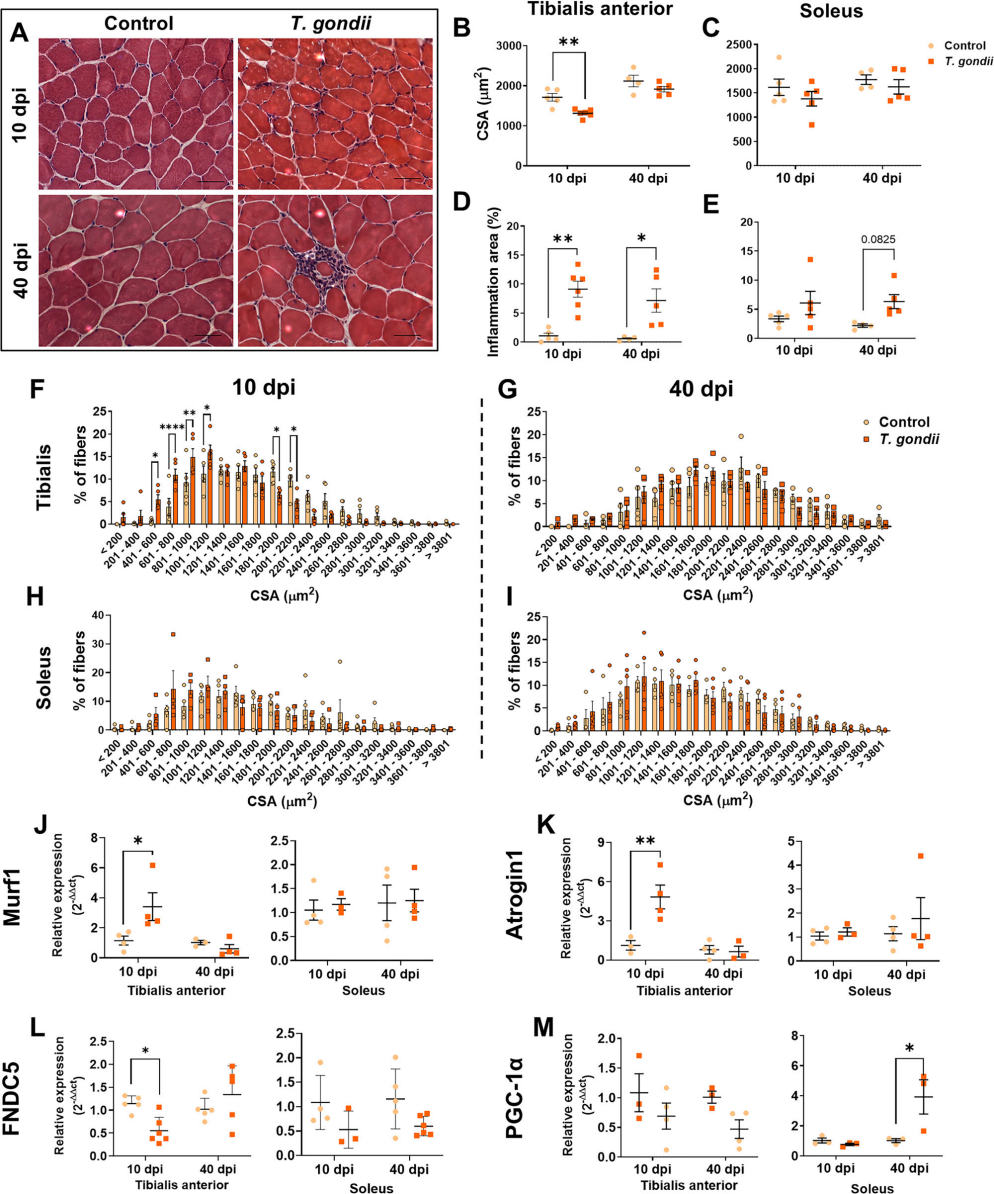
*T. gondii* infection leads to muscle atrophy. Hematoxylin-eosin staining of TA (**A**) muscle after 10 and 40 days, and SOL was used to measure CSA (**B**, **C**) and inflammation area (**D**, **E**). CSA of TA was decreased at 10 dpi and did not change at 40 dpi. The inflammation area of TA reached 9% 10 dpi and 7% 40 dpi. CSA fibers distribution of TA (**F**) showed that infected mice have smaller fibers after 10 days, especially until 1200 μm, and no changes were observed at 40 dpi (**G**). No changes were observed in fiber distribution in SOL (**H**, **I**). Murf1 (**J**), Atrogin1 (**K**), FNDC5 (**L**), and PGC-1α (**M**) expression was measured by RT-qPCR. *: *p* < 0.05, **: *p* < 0.01, ****: *p* < 0.0001. Two-Way ANOVA, Bonferroni’s post-test. Each dot in the graphs corresponds to an independent mice. Scale bar = 50 μm.

TA from infected mice had reduced fibers at 10 dpi (*p* < 0.01), which were restored to control levels at 40 dpi (Fig. 2A, B), whereas SOL CSA was unaltered (Fig. 2C). Inflammatory area was greater in infected TA tissue in both times evaluated reaching 9% and 7% of tissue area at 10 and 40 dpi, respectively (Fig. 2D). A trend to increase the inflammatory area at 40 dpi was observed in SOL, although not statistically significant (Fig. 2E). Accordingly, the fiber distribution showed a predominance of smaller fibers in infected TA muscle at 10 dpi, which was restored at 40 dpi (Fig. 2F–I).

Muscle atrophy markers Murf1 and Atrogin1 were strongly increased by infection in TA at 10 dpi, by 3-fold and 5-fold, respectively, whereas no changes were observed in SOL (Fig. 2J, K). Irisin is an exerkine^19,50^ and was shown to ameliorate the neuropathology of Alzheimer’s disease^28^. FNDC5, the irisin precursor, was reduced in TA following infection (Fig. 2L), although no changes were seen in SOL muscle or in serum irisin levels (*data not shown*). In the brain, Fndc5 expression was selectively reduced in the cerebellum of *T. gondii*-infected mice at 40 dpi and no changes were observed at the cortex and hippocampus regions (Supplementary Fig. 4). PGC-1α, a signaling hub protein involved in irisin expression and mitochondrial biogenesis^51^ showed no alteration in TA. However, PGC-1α had a 4-fold increase at 40 dpi in SOL (*p* < 0.05; Fig. 2M). Altogether, these results indicate that *T. gondii* infection dynamics and the onset of muscle pathology varies according to the muscle affected.

### Muscle fiber regeneration and composition are affected in acquired *T. gondii* infection

**Fig. 3:**
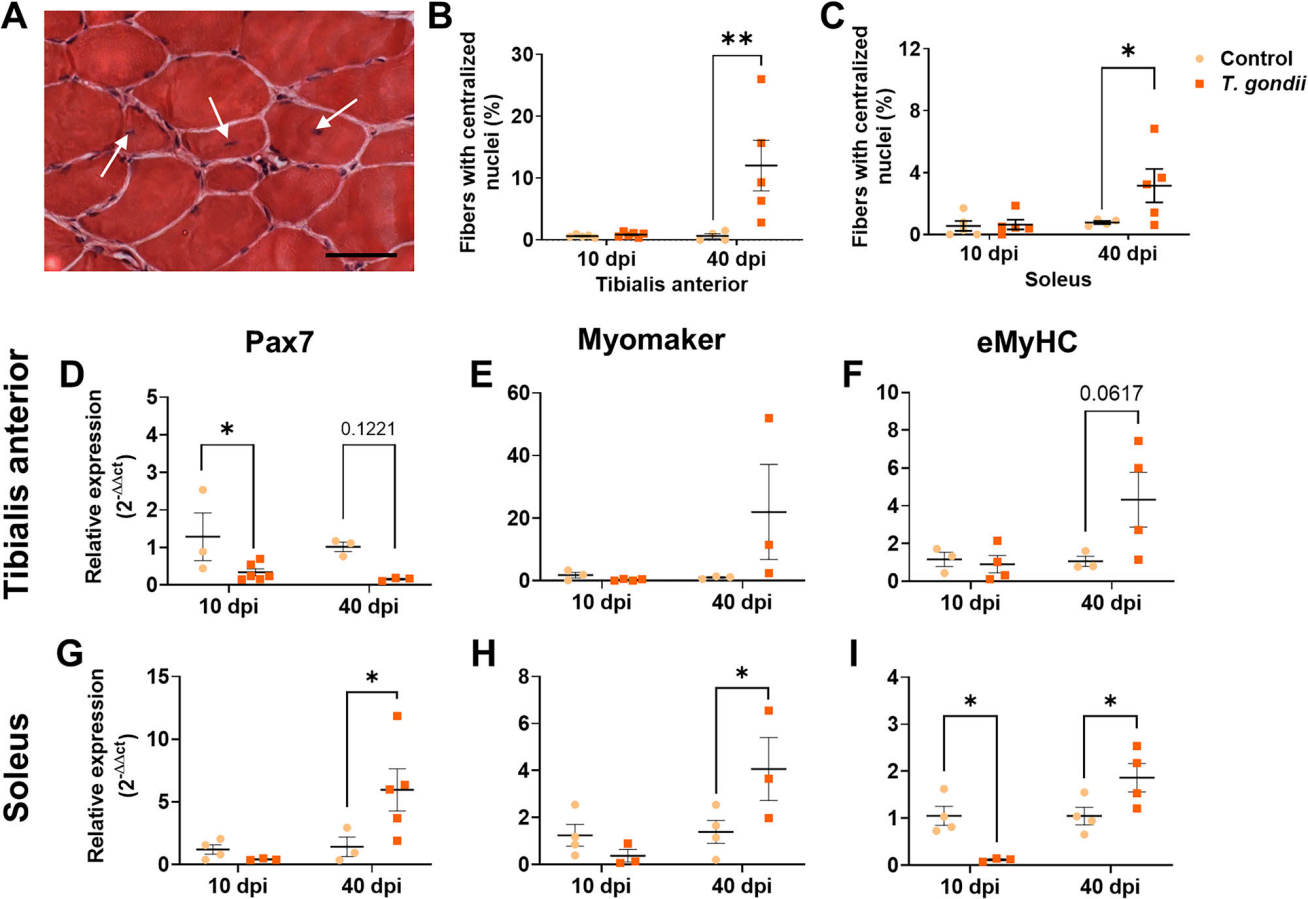
Effects of *T. gondii* acquired infection on skeletal muscle regeneration. **A** H&E representative image of *T. gondii*-infected TA muscle, with centralized nuclei (arrows), indicating regeneration process. Centralized nuclei fibers were increased in TA (**B**) and SOL (**C**) at 40 dpi. Regeneration markers Pax7 (**D**, **G**), Myomaker (**E**, **H**), and eMyHC (**F**, **I**) expression were measured by RT-qPCR in TA (**D**–**F**) and SO (**G**–**I**). *: *p* < 0.05, **: *p* < 0.01. Two-Way ANOVA, with Bonferroni’s post-test. Each dot in the graphs corresponds to an independent mice. Scale bar = 50 μm.

Regeneration following injury is an important feature of the SkM and relies on the activation of muscle-resident satellite stem cells^52^. Both TA and SOL muscles had an increased number of myofibers with centralized nuclei at 40 dpi, a hallmark of regenerating myofibers (^53^; Fig. 3A–C). Pax7 expression was decreased in TA muscles at 10 dpi and had a trend to decrease at 40 dpi (Fig. 3D). In SOL, however, there was an increase of Pax7 expression after 40 days (*: *p* < 0.05), indicating a possible late regenerative process (Fig. 3G). Myomaker expression, important for the myoblast fusion process^54^, was not altered in TA muscle (Fig. 3E) but was significantly increased in soleus muscle, at 40 dpi (Fig. 3H). Embryonic myosin (*Myh3*), expressed in regenerating myofibers^55^, was decreased at 10 dpi in the SOL muscle (Fig. 3I), followed by increased expression at 40 dpi in SOL and a tendency towards an increase in TA (*p* = 0.0617; Fig. 3F, I).

**Fig. 4:**
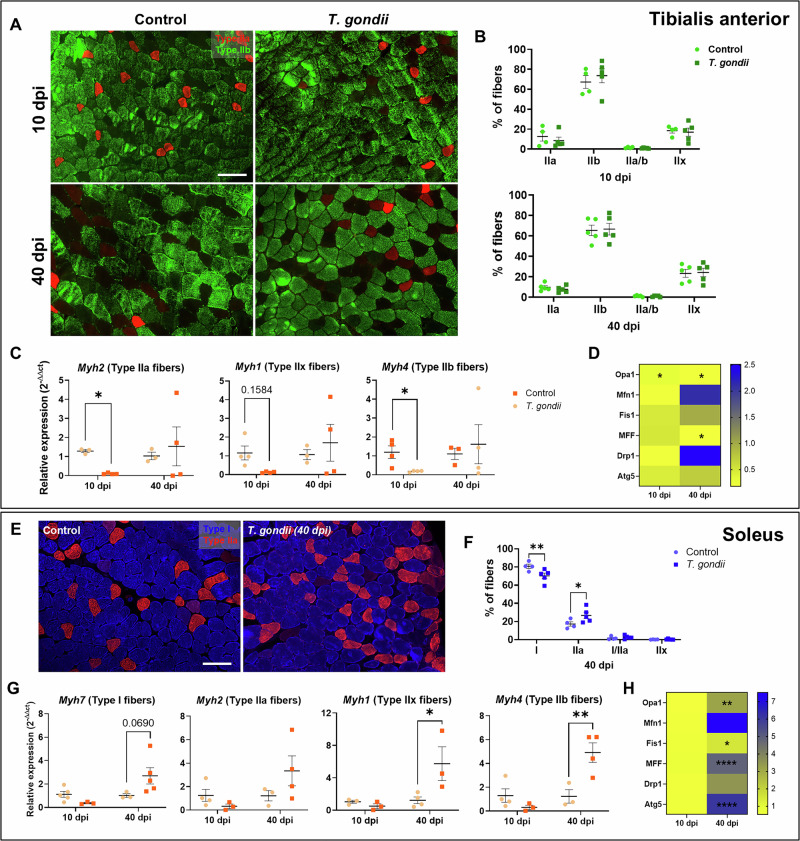
Fiber type composition is affected by *T. gondii*infection. Myosin heavy chain isotypes IIa (red) and IIb (green) were stained in TA (**A**), and their proportion was quantified 10 and 40 dpi (**B**, **C**). *Myh2* (type IIa), *myh1* (IIx), and *myh4* (IIb) expression were decreased or had a trend to increase in infected mice (**C**). **D** Heatmap showing mitochondria fusion (Opa1, Mfn1), fission (Fis1, MFF, and Drp1), and autophagy (Atg5) markers expression in TA, compared to control animals. **E** MyHC types I (in blue) and IIa (in red) were stained in SOL samples at 40 dpi. Type I fibers proportion was decreased with infection as Type IIa fibers increased (**F**). Expression of MyHC isotypes *myh7* (type I), *myh2, myh1,* and *myh4. Myh1* and *myh4* showed an increase 40 dpi in SOL (**G**). Mitochondria dynamic markers were assessed in SOL at 10 and 40 dpi (**H**). *: *p* < 0.05, **: *p* < 0.01, ****: *p* < 0.0001. Two-Way ANOVA, Bonferroni’s post-test. Each dot in the graphs corresponds to independent mice. Scale bar: 100 μm.

Given the distinct characteristics of fiber composition between oxidative (SOL) and glycolytic (TA) muscles, we assessed the different fiber type ratios after infection. As expected, TA had a predominance of IIb and IIx type fibers^56^. A slight increase in the number of IIb fibers was observed at 10 dpi (*p* = 0.058; Fig. 4A, B), whereas no changes were observed at 40 dpi (Fig. 4B). We evaluated the expression of myosin isoforms that compose type IIa (*Myh2*), IIx (*Myh1*), and IIb (*Myh4*) fibers, and found that in the TA muscle, all three isoforms were downregulated at 10 dpi and restored at 40 dpi (Fig. 4C). *T. gondii* is known to differentially infect and form cysts according to the host cell metabolic state, and this holds true for SkM fibers^12^; mitochondrial quality control is important for SkM metabolic status^57,58^. Therefore, we evaluated markers of mitochondrial plasticity in infected TA muscle. Mitochondrial fusion-related OPA1 transcripts were significantly down-regulated both at 10 and 40 dpi (0.3- and 0.18-fold, respectively), whereas fission marker MFF was down-regulated solely at 40 dpi (0.18-fold, Fig. 4D).

As an oxidative muscle, SOL presented a different composition compared to TA muscles, including the massive presence of type I fibers, which corresponded to 81% of the total fibers (Fig. 4E), in accordance with previous evidence^59^. After 40 days of infection, SOL muscles had a significant decrease in type I fiber density (70% in infected mice) and an increase in type IIa fibers (26.6% in infected mice, versus 17% in controls, Fig. 4E, F). Expression of different MyHC isoforms in SOL muscle was not affected at 10 dpi but was either increased—*myh1* and *myh4*—or tends to increase at 40 dpi—*myh7* and *myh2* (Fig. 4G). Regarding mitochondrial plasticity markers, SOL exhibited an opposite response to infection as compared to TA, with a significant increase in OPA1, MFF, and Fis1 expression, as well as an increase in mitophagy marker Atg5 (Fig. 4H). These results indicate important changes in fiber composition and regeneration depending on the fiber type.

### *T. gondii* infection elicits an innate immune response leading to IL6-STAT3 axis activation in the SkM

**Fig. 5:**
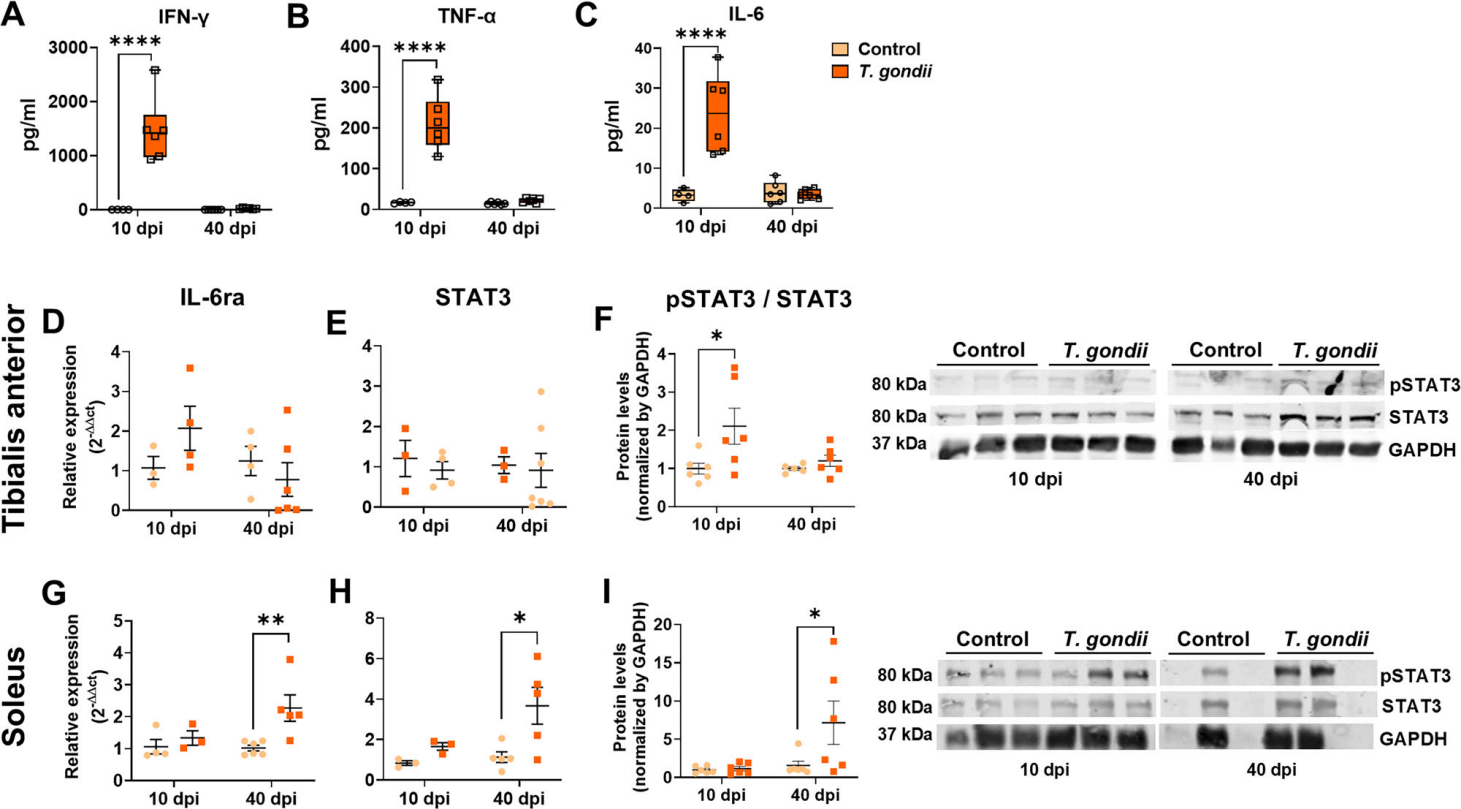
Systemic and local muscle inflammatory profile of *T. gondii* infection in mice. Cytokine response to *T. gondii* infection and IL-6 pathway. The IFN-γ (**A**), TNF-α (**B**), and IL-6 (**C**) serum levels in *T. gondii*-infected mice were evaluated with CBA. IL-6ra (**D**) and STAT3 (**E**) mRNA expression did not change in TA, but pSTAT3/STAT3 was increased at 10 dpi (**F**). In SOL, IL-6ra and STAT3 (**G**, **H**) mRNA expression was increased at 40 dpi, as well as STAT3 phosphorylation levels (**I**). *: *p* < 0.05, **: *p* < 0.01, ****: *p* < 0.0001. Two-Way ANOVA, Bonferroni’s post-test. Each dot in the graphs corresponds to an independent mice.

Systemic inflammatory response in female SW mice following *T. gondii* infection is composed of increased serum levels of IL-6, IFN-γ, and TNF-α at 10 dpi, returning to control levels at 40 dpi (Fig. 5A–C). IL-2, IL-4, IL-10, and IL-17 levels were not significantly altered at either time point (Fig. S2). IL-6 signaling is widely known for its muscle atrophy effect^60^, and this influence has been demonstrated in other models of infection^61^. IL-6 receptor (IL6ra) and STAT3 expression remained unaltered in TA muscle (Fig. 5D, E). However, STAT3 phosphorylation was found to be significantly increased at 10 dpi (Fig. 5F). Conversely, *IL6ra* and *Stat3* transcripts were found significantly increased in SOL muscle at 40 dpi, as well as pSTAT3 levels (Fig. 5G–I). Thus, *T. gondii* resulted in an inflammatory profile in mice, particularly in IL-6-STAT3 signaling mechanisms.

### Endurance physical exercise is protective from *T. gondii*-induced muscle wasting

**Fig. 6:**
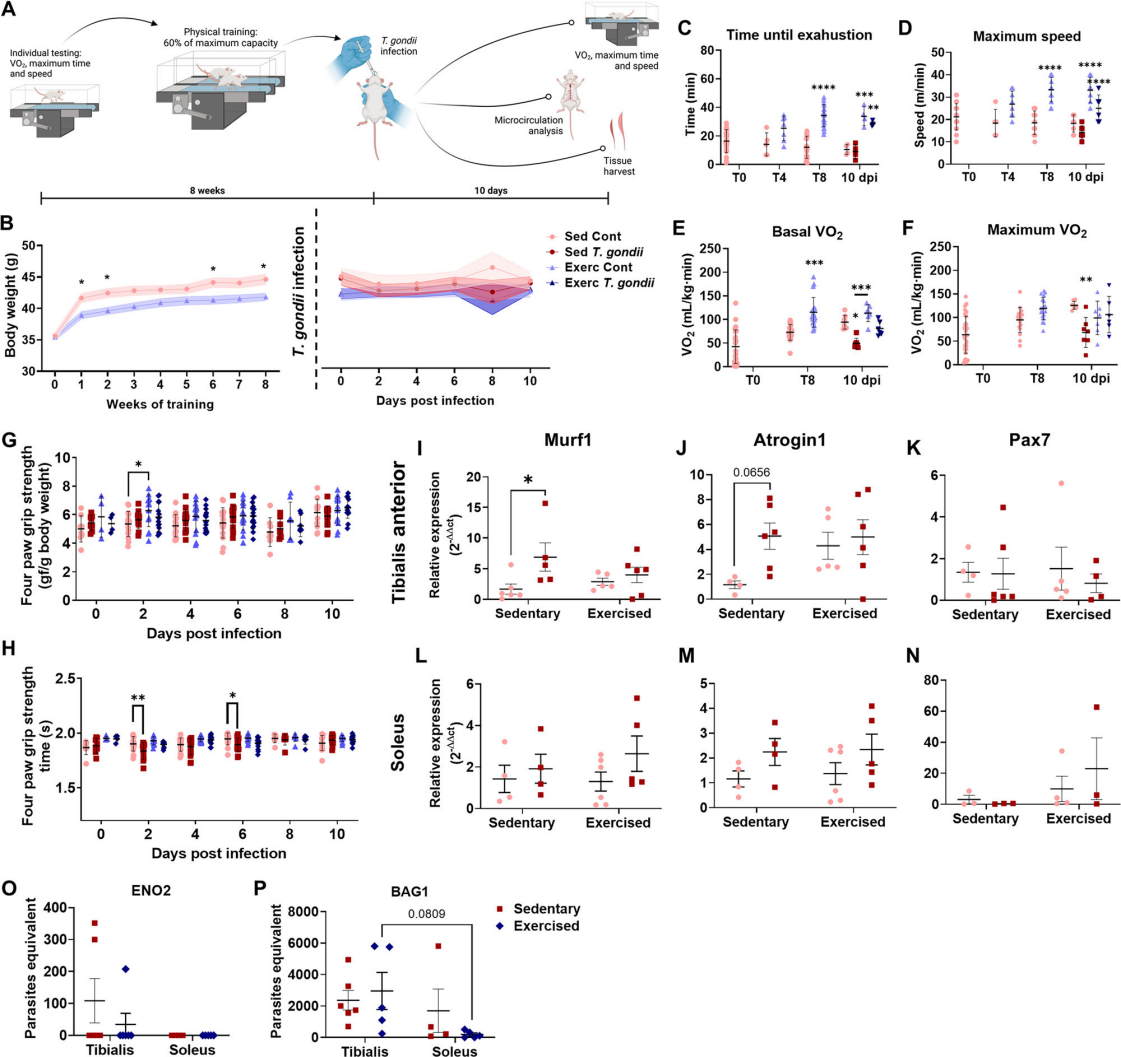
Physical exercise training prevents metabolic effects, functional loss, and muscle atrophy. **A** Experimental design of physical training protocol. Created in BioRender. Adesse, D. (2025) https://BioRender.com/72ra8nk. **B** Body weight started to decrease after the first week of exercise training. After infection (**B**), no significant changes were noticed in body weight. Time until exhaustion (**C**) and maximum speed (**D**) were recorded through the training and after *T. gondii* infection. T0, T4, and T8 indicate time points (in days) following physical training. Basal oxygen consumption (VO_2_) and maximum (VO_2max_) were decreased only in sedentary-infected animals (**E**, **F**, respectively). Grip strength (**G**) was not altered by infection, but increased in exercised animals; grip strength time (**H**) was decreased only in sedentary-infected animals. Murf1 expression in TA was increased in sedentary animals but not in exercised ones (**I**). Atrogin1 and Pax7 expressions were not altered significantly (**J**, **K**). In SOL, no changes were observed (**L**–**N**). *T. gondii* tachyzoites ENO2 expression was detected only in a few samples of TA muscles (**O**), as bradyzoites BAG1 expression was higher in TA (**P**). *: *p* < 0.05, **: *p* < 0.01, ***: *p* < 0.001, ****: *p* < 0.0001. Two-Way ANOVA, Bonferroni’s post-test. Each dot in the graphs corresponds to an independent mice.

We next interrogated whether physical exercise could be protective in Toxoplasma-induced pathology. For this purpose, we subjected SW male mice to an 8-week protocol of treadmill endurance exercise (Fig. 6A) followed by *T. gondii* infection for 10 days, when major histopathological damage was observed. As expected, sedentary mice had a more pronounced body weight gain than exercised ones, although, in contrast to female mice, no weight loss was observed in sedentary male mice following *T. gondii* infection (Fig. 6B). SOL, but not TA, muscle mass was increased after exercise regimen in uninfected mice, whereas *T. gondii*-infected animals did not show this increase in muscle mass (Fig. S3A, B). Interestingly, *T. gondii* infection led to a decrease in cervical brown adipose tissue, with no changes in white adipose tissue, in sedentary mice (Fig. S3C–E). Maximum speed and time until exhaustion in the treadmill were increased at the end of the training protocol (Fig. 6C, D). Basal VO_2_ was also increased after 8 weeks of exercise. *T. gondii* infection led to a decrease in VO_2max_ in sedentary mice, but physical exercise prevented this effect (Fig. 6E, F).

Sedentary-infected male mice had decreased grip time after 2 and 6 dpi, but no effect on relative grip strength peak (Fig. 6G, H), whereas exercised-infected ones did not display significant differences in grip strength time after infection (Fig. 6H). Atrophy marker Murf1 was up-regulated in sedentary-infected TA muscles, similarly to the observed in females. However, exercised-infected mice showed Murf1 levels matching uninfected controls (Fig. 6I). Atrogin1 and Pax7 transcripts remained unaltered in TA muscles in infected groups as compared to their respective controls (Fig. 6J, K). No changes in atrogenes were observed in SOL muscle (Fig. 6L–N). Mitochondrial plasticity genes were increased in TA in exercised mice and *T. gondii* infection led to reduced Opa1, Mfn1, Fis1 and Dnml1 (Drp1) expression, whereas the SOL muscle showed less pronounced effects (Supplemental Fig. 4). *Fndc5* expression was consistently reduced in TA samples of *T. gondii*-infected mice, in both sedentary and exercised groups, and no changes were observed in SOL tissue as well as in mouse serum (Supplementary Fig. 5D–F). PGC-1α mRNA levels were unaltered in both TA and SOL tissues across all experimental groups (Supplementary Fig. 5G, H). To assess if the exercise protective effects could be caused by modulation of *T. gondii* replication in the host, we evaluated tachyzoite and bradyzoite markers (*eno2* and BAG1, respectively) in both TA and SOL muscles. In males, the BAG1 signal was widely detected in TA samples, whereas the SOL samples had a significantly smaller parasite load. Physical exercise had no effect on either tachyzoite or bradyzoite loads in SkM (Fig. 6O, P).

**Fig. 7:**
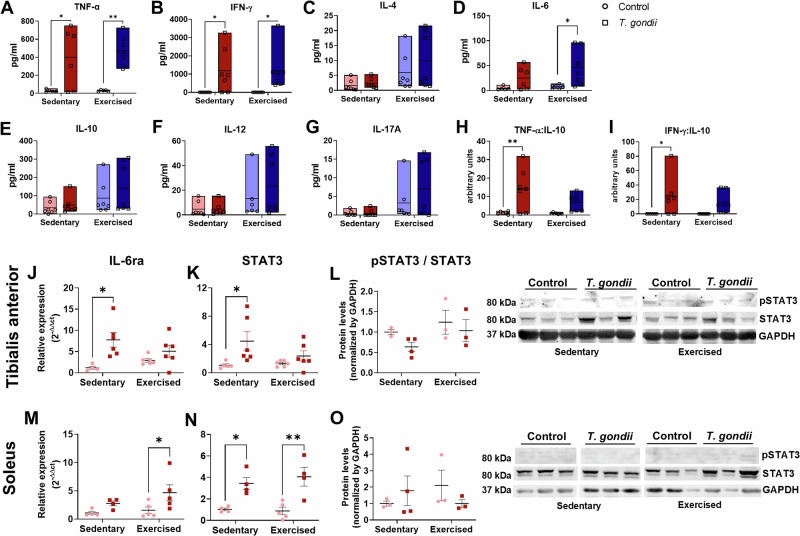
Physical exercise modulates immune response to *T. gondii* infection. Serum levels of TNF-α (**A**), IFN-γ (**B**), IL-4 (**C**), IL-6 (**D**), IL-10 (**E**), IL-12 (**F**), and IL-17A (**G**) showing an increase of TNF-α and IFN-γ secretion in both infected groups, whereas IL-6 levels were higher in exercised-infected mice. Pro and anti-inflammatory cytokines ratio was calculated using TNF-α/IL-10 (**H**) and IFN-γ / IL-10 (**I**) values from each animal. IL-6ra (**J**) and STAT3 (**K**) expression in TA was increased only in sedentary-infected mice, and STAT3 phosphorylation was not affected (**L**). In SOL, IL-6ra expression was increased in exercised-infected animals (**M**), and STAT3 mRNA was increased in both infected groups (**N**). STAT3 phosphorylation was not changed in SOL (**O**). *: *p* < 0.05, **: *p* < 0.01. Two-Way ANOVA, Bonferroni’s post-test. Each dot in the graphs corresponds to an independent mice.

Similar to what was observed in sedentary female mice, *T. gondii* infection resulted in an increase in serum TNF-α and IFN-γ in sedentary male mice at 10 dpi, as well as in TNF-α, IFN-γ, and IL-6 serum levels in exercised animals, whereas no change was observed for IL-4, IL-10, IL-12, and IL-17A (Fig. 7A–G). Despite the increase in pro-inflammatory cytokines observed in infected groups, we evaluated the balance between TH1/TH2 response as a function of the TNF-α/IL-10 and IFN-γ/IL-10 ratios (Fig. 7H, I). Whereas in sedentary-infected mice, both the TNF-α/IL-10 and the IFN-γ/IL-10 ratios were significantly elevated, exercised-infected mice had results comparable with uninfected controls (Fig. 7H, I). In the TA muscle, IL-6ra and STAT3 transcripts were significantly increased in sedentary-infected mice, whereas no change was observed in exercised-infected ones (Fig. 7J-, K), although pSTAT3 remained unaltered in infected male mice (Fig. 7L). The SOL muscle showed a different response, with increased IL-6ra expression in exercised-infected but not in sedentary-infected mice (Fig. 7M), and increased STAT3 gene expression in infected mice (Fig. 7N), although no change was observed in pSTAT3 (Fig. 7O). Thus, these results suggest an important role of exercise in regulating the inflammatory response against infections.

### Toxoplasma-induced microvascular damage in the SkM and in the brain is prevented by physical exercise

**Fig. 8:**
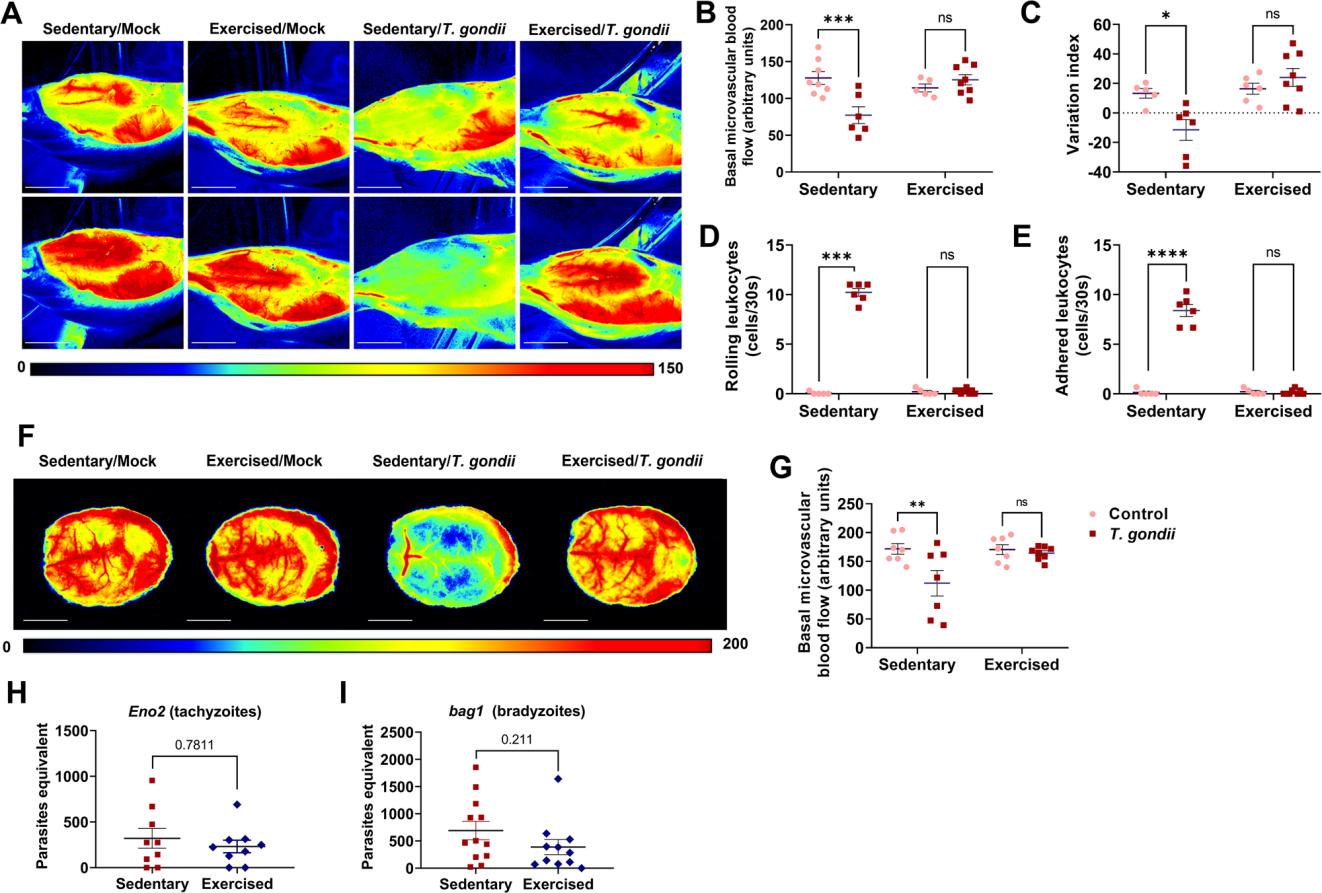
Physical exercise protects muscle and brain microcirculation in infected mice. Laser Speckle representative images of the TA muscle at basal levels (upper panels) and after acetylcholine administration (lower panels) are shown in (**A**). Scale bar = 0.5 cm. Microvascular blood flow in TA is reduced by infection in sedentary animals and not in exercised-infected (**B**). Following acetylcholine stimulus, sedentary-infected animals responded with vasoconstriction, whereas exercised-infected showed vasodilation response, similar to uninfected controls (**C**). Leukocyte-endothelium interaction, assessed by the numbers of rolling (**D**) and adhered (**E**) leukocytes, also showed a protective role of physical exercise. Brain microvascular blood flow was analyzed by LSCI (**F**). Scale bar = 0.5 cm. Infection leads to decreased blood flow only in sedentary animals (**G**). Brain cortex parasite load, assessed by RT-qPCR for ENO2 (tachyzoites, **H**) and bag1 (bradyzoites, **I**), showed no effect of physical exercise on parasitism. *: *p* < 0.05, **: *p* < 0.01, ***: *p* < 0.001, ****: *p* < 0.0001. Two-Way ANOVA, Bonferroni’s post-test. Each dot in the graphs corresponds to independent mice.

Our group demonstrated that *T. gondii* acquired infection leads to a sustained cerebral capillary rarefaction, accompanied by increased leukocyte-endothelium interaction and endothelial dysfunction^42^. Herein, we observed that *T. gondii* led to a significant reduction in blood flow in the SkM of sedentary infected mice compared to sedentary controls, as shown by LSCI, whereas exercised-infected mice had blood flow levels comparable to uninfected controls (Fig. 8A, B). Endothelial function, assessed by measuring vasodilation response to acetylcholine^62^, was disrupted in the infected sedentary mice SkM; however, physical exercise protected the tissue (Fig. 8C).

Leukocyte-endothelium interaction was significantly increased in sedentary-infected mice TA, compared to respective controls, while exercised-infected mice showed no signs of leukocyte recruitment (Fig. 8D, E). Moreover, we observed that in sedentary male SW mice, similar to what we described in females^42^, *T. gondii* infection leads to reduced cerebral blood flow (Fig. 8F, G), which was not observed in exercised-infected animals. Finally, we observed that physical exercise had no effect in brain parasite load, as shown by RT-qPCR (Fig. 8H, I).

## Discussion

We investigated the effects of experimental *T. gondii* infection on metabolic divergent SkM of mice. The infection led to differential damage in glycolytic and oxidative fibers, with faster onset in glycolytic muscles, and induced a slow-to-fast transition in the oxidative fibers. Some injuries or pathologies can promote more damage in one fiber type than the other^63,64^. Glycolytic fibers can be more affected in cachexia, as type I fibers are more compromised after the denervation process^63^. In Duchenne’s muscle dystrophy (DMD) patients, fast-type fibers are more susceptible to degeneration. In fact, treatments that induce an oxidative phenotype shift stimulation protect the muscle from DMD damage^65^. Impacts in mitochondrial respiration in glycolytic, but not oxidative, muscle tissues were seen in mdx mice, a model for DMD^66^. Herein, we observed a differential increase in atrogenes expression in TA (Murf1 by 3-fold and Atrogin by 5-fold) but not in SOL in early infection, leading to a pronounced atrophy process at this time-point. Not only smaller fibers were observed in *T. gondii* infected animals, but a greater inflammatory area in TA skeletal muscle slices. Inflammatory cells and its cytokines may directly contribute to the destruction of muscle fibers. Prolonged maintenance of the inflammatory phase can not only delay the regenerative process but also activate pathways of atrophy and muscle fiber destruction, including activation of atrogenes expression^67^. This systemic inflammatory process could lead to decreased resistance observed in functional tests.

Regarding the infection dynamics, it is known that stress factors, such as NO, trigger tachyzoite to bradyzoite conversion^68^. Rahman and collaborators^12^ demonstrated the importance of cell redox status to *T. gondii* stage conversion. Increased ROS levels led to increased bradyzoite stage conversion in SkMCs, whether they are naturally present (as showed in myotubes vs myoblasts) or experimentally induced^12^. *T. gondii* infection can induce exacerbated NO production, which can lead to oxidative stress, cellular dysfunction, apoptosis, and tissue damage, been especially important in *T. gondii* neuropathology^69^. The selective parasite burden observed here suggests that TA damage is induced by the parasite presence and the associated local damage response, whereas SOL damage is caused by systemic inflammatory mediators, resulting in a slower onset. It is also important to mention the differences observed in *T. gondii* infection in males and females. At 10 dpi, the time point with the most disease-induced features, BAG1 expression in TA was more than twice in sedentary males, compared with females (Fig. S3H). This could explain some differences in the infection, such as grip strength and time. Notably, the impact on grip strength was evident shortly after infection in males, whereas females exhibited these differences after a longer period.

We described a slow-to-fast transition in SOL at 40 dpi and a trend towards a shift in TA. A known inductor of slow-to-fast fiber shift is ROS accumulation, which can be triggered by parasitic infections, including *T. gondii*^70,71^. These data corroborate the results described herein, with a slow-to-fast fiber transition in *T. gondii* infected mice, similar to what is observed in DMD patients. Although there is a slow-to-fast transition in *T. gondii* infected mice, we did not identify a differential regulation of any MyHC isoforms in mRNA expression at the timepoints evaluated. The infection led to reduced MyHC expression in TA 10 dpi, which agrees with our previous results in C2C12 cells^35^. In SOL, expression of all MyHC isoforms seems to be increased 40 dpi. Notably, MyHC is one of the main targets of ubiquitination by E3 ubiquitin ligases, and its degradation is Murf1-dependent^72^. As those atrogenes were positively modulated by *T. gondii* infection, this could represent a regenerative effect.

PGC-1α is a regulator of mitochondrial biogenesis found in SkM and brown adipose tissue^51^. Some studies demonstrated that PGC-1α may play a protective role in oxidative muscles^63,73,74^. It prevents muscle atrophy by inhibiting the transcriptional activity of FoxO, which then suppresses Murf1 and Atrogin1^75^. Herein, PGC-1α was highly increased in infected mice at 40 dpi. Given its effect in slow fiber formation, we suggest that it may play a compensatory role in restoring SOL fiber composition.

Endurance exercise (otherwise known as aerobic or cardiorespiratory) consists of a continuous series that can be performed at varying intensities^76^. Increased aerobic capacity can be a valuable tool to improve respiratory diseases, including COVID, due to its antioxidant action, restoration of lung capacity and elasticity, and enhancement of the immune response^77^. Moderate-intensity treadmill exercise can induce an M2 macrophage profile, thus promoting reduced inflammation in the adipose tissue of obese animals and facilitating healing [^78,79^]. Our findings showed a different profile in pro- and anti-inflammatory cytokine levels in exercised animals and, notably, a modulation of the cytokine ratio in the exercised-infected groups, possibly due to increased basal levels of anti-inflammatory IL-4, IL-10, and regulatory IL-17A in exercised mice as compared to sedentary. Considering that one of the *T. gondii* infection characteristics is immune modulation with a predominance of M1 macrophages in the SkM^49^, exercise may shift this phenotype, promoting balance and tissue restoration in the damaged area. Exercise can also influence oxygen consumption in two main aspects: improving oxygen delivery through blood and cardiovascular function, and the adaptations of the tissue microenvironment, such as increased tissue capillarization and mitochondrial content^80^.

*T. gondii* infection drastically reduced the capillary blood flow and endothelial integrity, factors directly related to oxygen consumption. We previously demonstrated severe brain vascular damage caused by *T. gondii* infection^42^. Herein, we also observed this damage and showed for the first time that physical exercise can prevent infection-induced cerebral and muscle vascular loss.

We interrupted physical training immediately after *T. gondii* infection to prevent a possible exacerbation of the acute phase of the disease. This strategy may have masked some of the beneficial effects of exercise, such as the stimulation of irisin and PGC-1α, which are rapidly upregulated^81–83^ but return to baseline levels within a few days after the stimulus cessation (Supplementary Fig. 5D–H). Despite this interruption, this exercise regimen was shown protective to cerebral and muscular microvasculature. Importantly, as expected, exercised uninfected mice showed a significant increase in SOL muscle mass, whereas in *T. gondii*-infected animals, this effect was abrogated. Given that the physical exercise protocol was suspended following infection, it is likely that some degree of muscle wasting or atrophy is still taking place in the SOL muscle.

Muscle parasite burden was not affected following exercise, in agreement with previous reports^84^. The suppression of several *T. gondii*-induced damages in exercised animals shows the importance of immune regulation, especially during parasite infections. Overall, our results support the notion that physical exercise was effective in preventing *T. gondii*-related damage, not only in glycolytic muscles, more affected, but importantly in brain microcirculation and systemic inflammation.

## Supplementary information

Supplementary Information

Supplementary Data 1

Gating strategy for CBA analysis

Description of Additional Supplementary Files

Reporting Summary

## Data Availability

All data supporting the findings of this study are available within the paper and its Supplementary Data file. Supplementary Figs. 6 and 7 contain the original, unprocessed blot images presented in Figs. 5 and 7.
