# Supplementary material for: Physical exercise protects against Toxoplasma gondii infection-induced muscle atrophy and microvascular rarefaction
Source: Commun Biol. 2026 Mar 10;9:562. doi: 10.1038/s42003-026-09810-9 (PMC13103317; doi:10.1038/s42003-026-09810-9)
Supplement: Supplementary file 1 — Supplementary Information [file 42003_2026_9810_MOESM1_ESM.pdf]

Supplementary figures

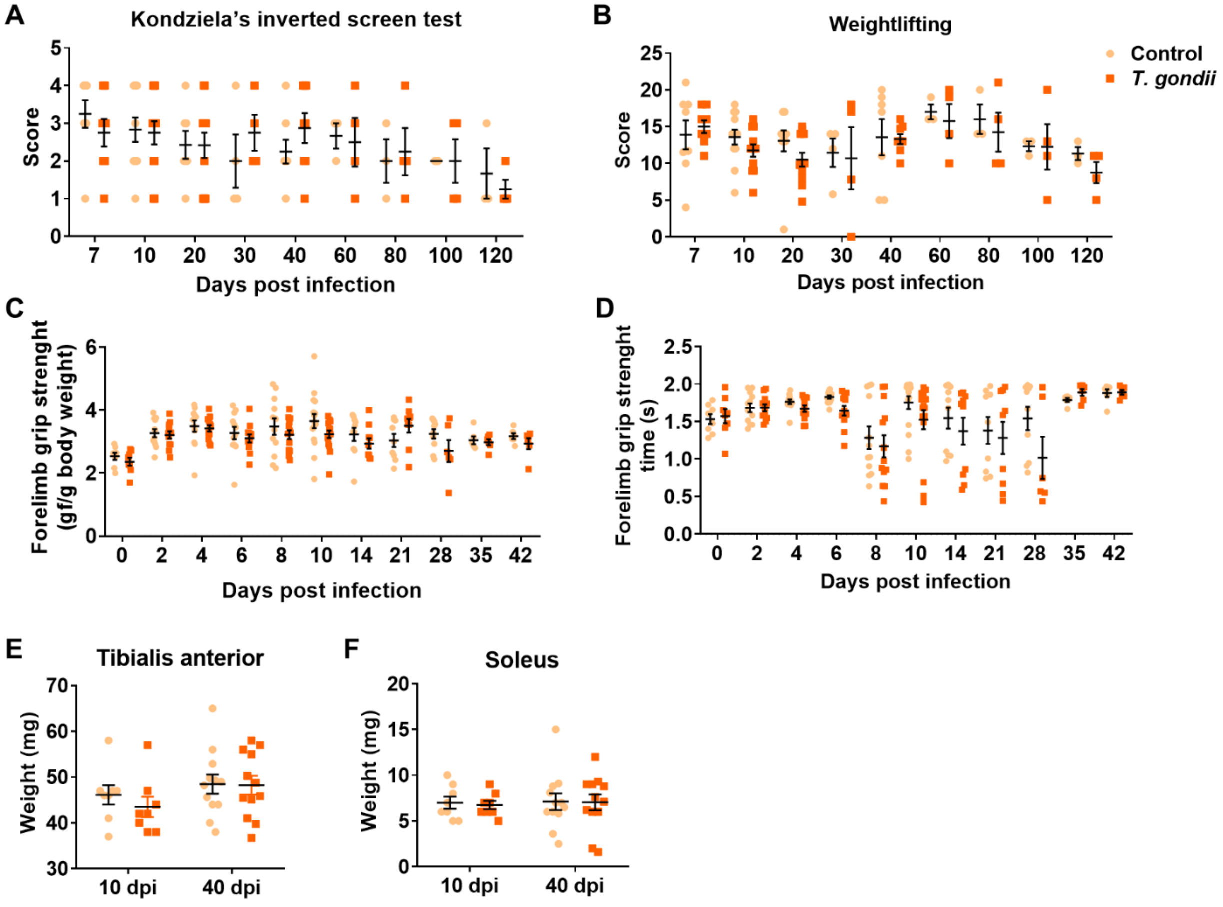

Supplementary Figure 1. Kondziela and weightlifting functional tests. A: Kondziela's inverted screen test was performed from the first week of infection, followed by 10 dpi, until 40 dpi in 10 days interval, and from 40 to 120 dpi each 20 days. Neither Kondziela's nor weightlifting (**B**) tests were modulated by *T. gondii* infection in females. **C**: In addition to four paw, grip strength test was also performed with the forelimbs, with no changes in strength peak or time (**D**). TA (**E**) and SOL (**F**) muscles weight did not change with infection.

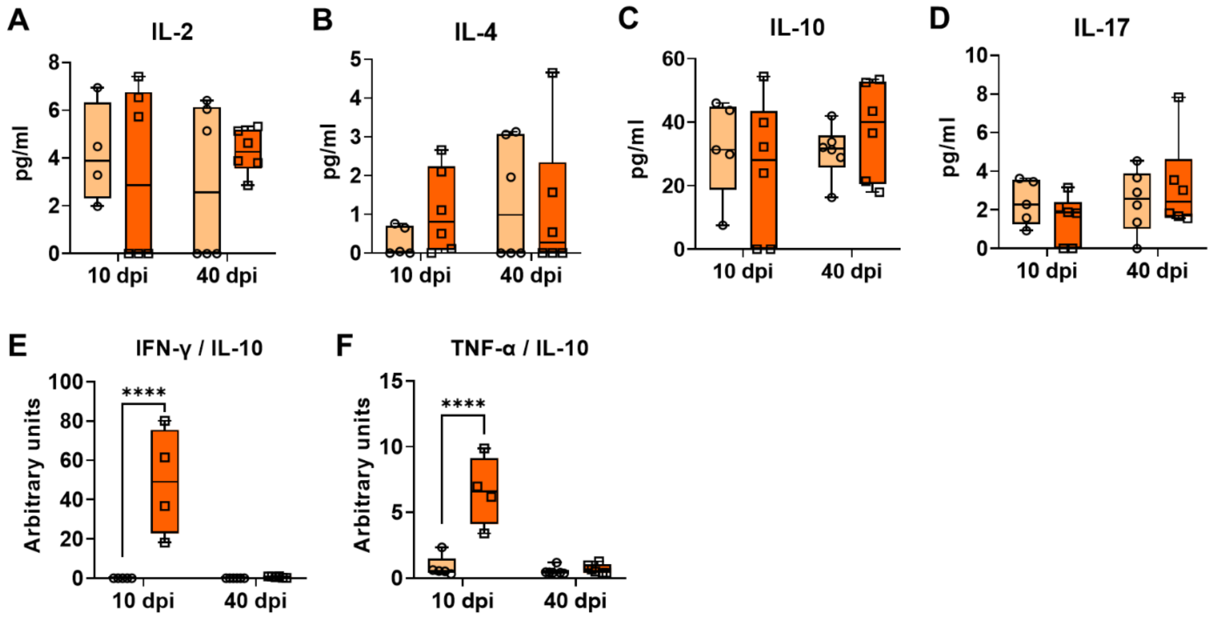

Supplementary Figure 2. Serum cytokine concentration in *T. gondii*-infected female mice. IL-2 (**A**), IL-4 (**B**), IL-10 (**C**) and IL-17 (**D**) secretion in response to *T. gondii* infection were evaluated with CBA. Pro and anti-inflammatory cytokines ratio was calculated using TNF-α / IL-10 (**E**) and IFN-γ / IL-10 (**F**) values showing an increase in 10 dpi. ****: $p<0.0001$. Two-Way ANOVA, Bonferroni's post-test.

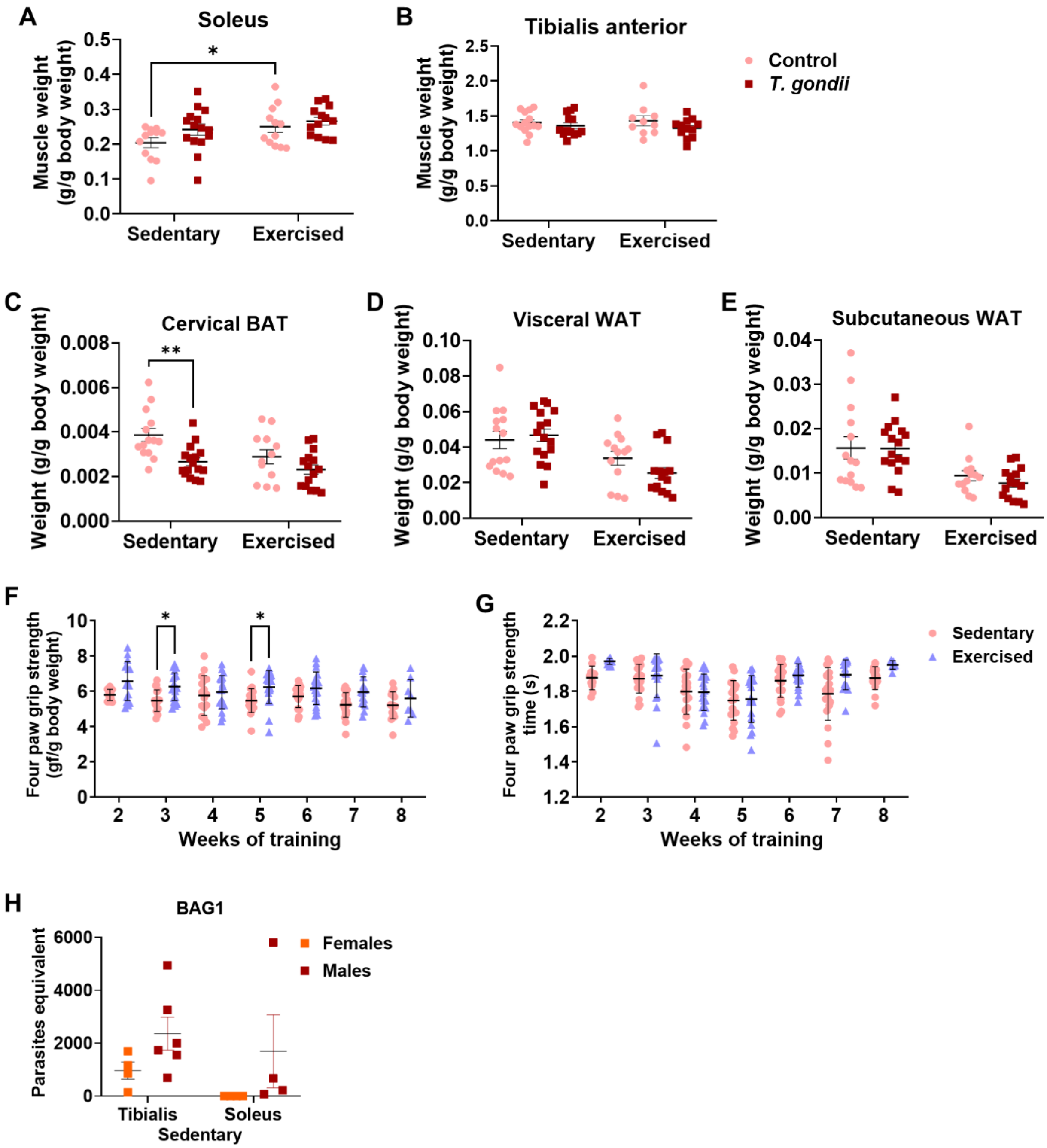

Supplementary Figure 3. Muscle and adipose tissues weight. SOL muscle weight was increased with physical exercise (A) and no changes were observed in TA muscle (**B**). Cervical brown adipose tissue (BAT) weight was decreased in sedentary infected mice (**C**) and no changes were observed in visceral and subcutaneous white adipose tissues (WAT, **D** and **E**, respectively).

Muscle and adipose tissues weight were normalized by each animal body weight. **F**: Four paw grip strength peak was higher in exercised animals from the third week of training, as grip strength time did not change (**G**). *: $p < 0.05$, **: $p < 0.01$ Two-Way ANOVA, Bonferroni’s post-test. **H**: Parasite load in TA and SOL was determined by RT-qPCR for BAG1 (bradyzoites) expression to compare infectivity of sedentary males and females at 10 dpi. Each dot in graphs corresponds to independent mice.

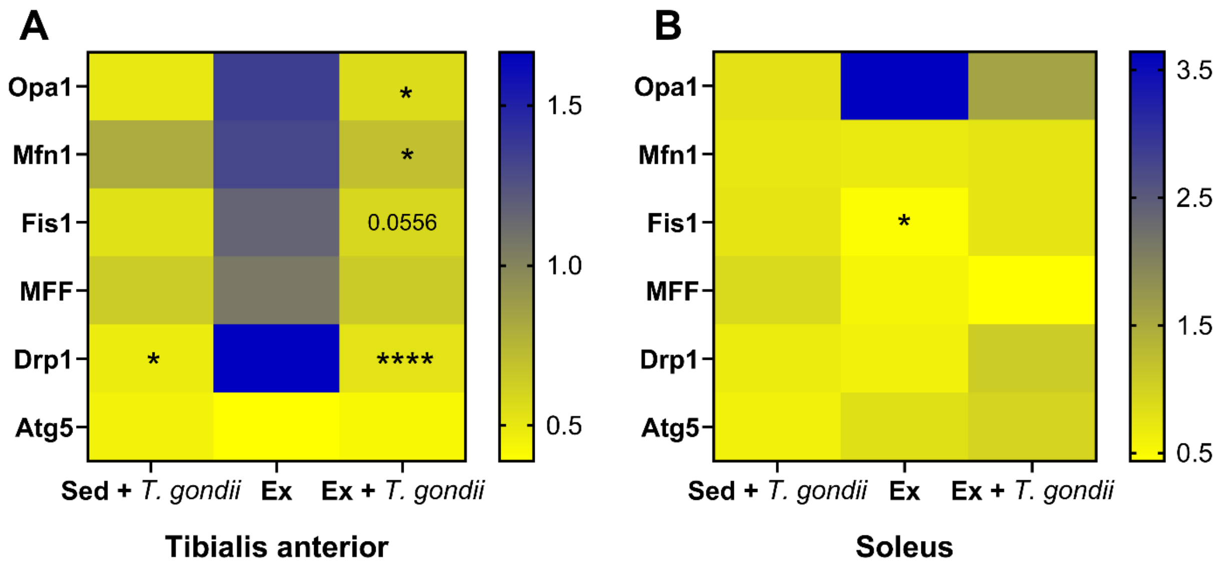

Supplementary Figure 4. Mitochondrial genes panel in exercised mice. Heatmaps showing mitochondria fusion (Opa1, Mfn1), fission (Fis1, MFF and Drp1) and autophagy (Atg5) markers expression in TA (**A**) and SOL (**B**). Values were normalized to uninfected sedentary group. OPA1, Mfn1, Fis1 and Drp1 were down-regulated in exercised-infected mice in TA. *: p<0.05, ****: p<0.0001, Two-Way ANOVA with Bonferroni post-test. Each dot in graph corresponds to an independent animal.

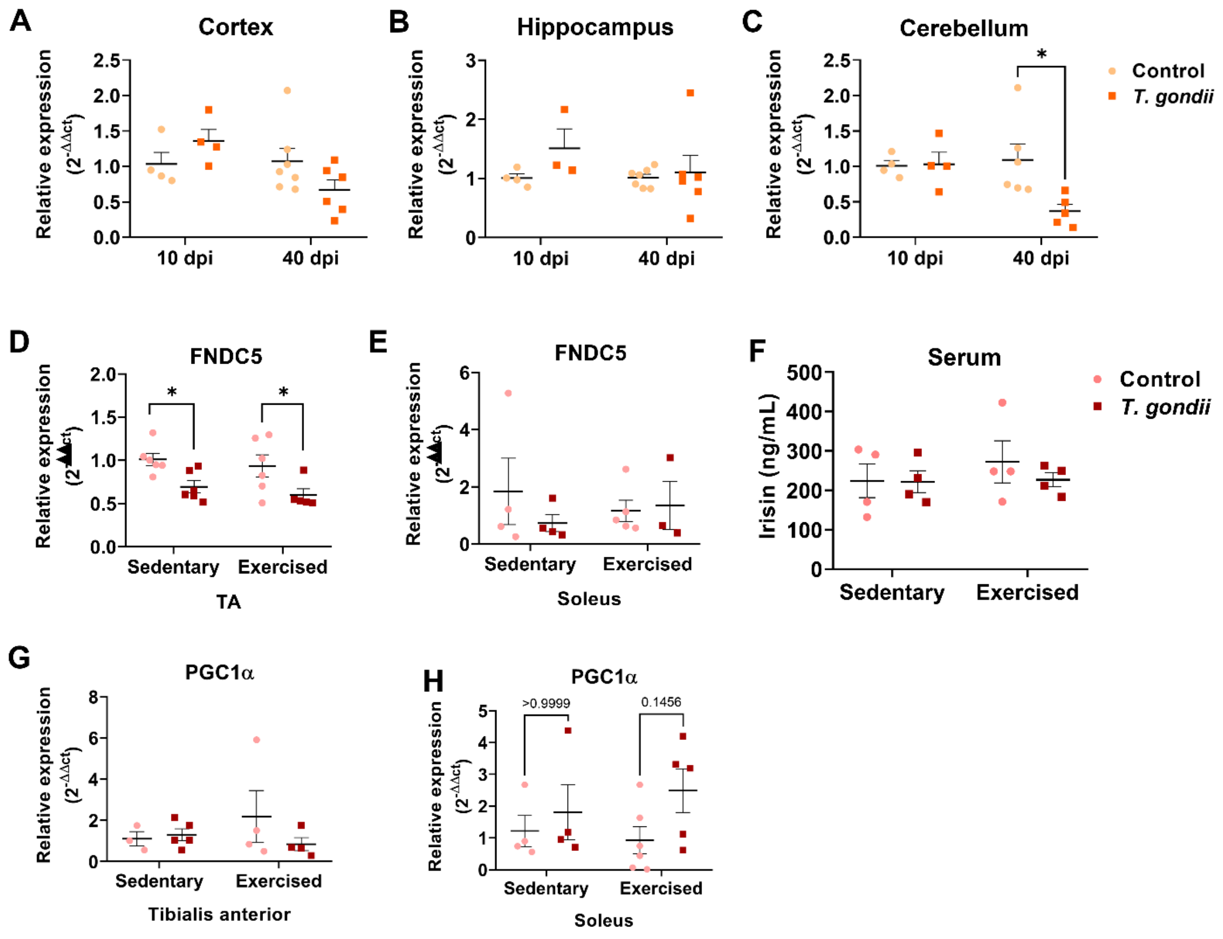

Supplementary Figure 5. Irisin expression in exercised animals. Brain cortex (**A**), hippocampus (**B**) and cerebellum (**C**) FNDC5 expression was assessed in female mice following *T. gondii* infection. *Fndc5* transcripts are decreased in cerebellum of infected mice at 40 dpi. FNDC5 expression in TA (**D**) and SOL (**E**) of sedentary or exercised male mice showed a significant reduction in infected animals in TA and no change in SOL muscle. Serum irisin levels were measured by ELISA and showed no change following infection (**F**). PGC-1α, that can be correlated with irisin production, had no change in expression in TA (**G**) or SOL (**H**) in infected mice, regardless of physical exercise regimen. *: $p<0.05$. Two-Way ANOVA, Bonferroni's post-test. Each dot in graphs corresponds to independent mice.

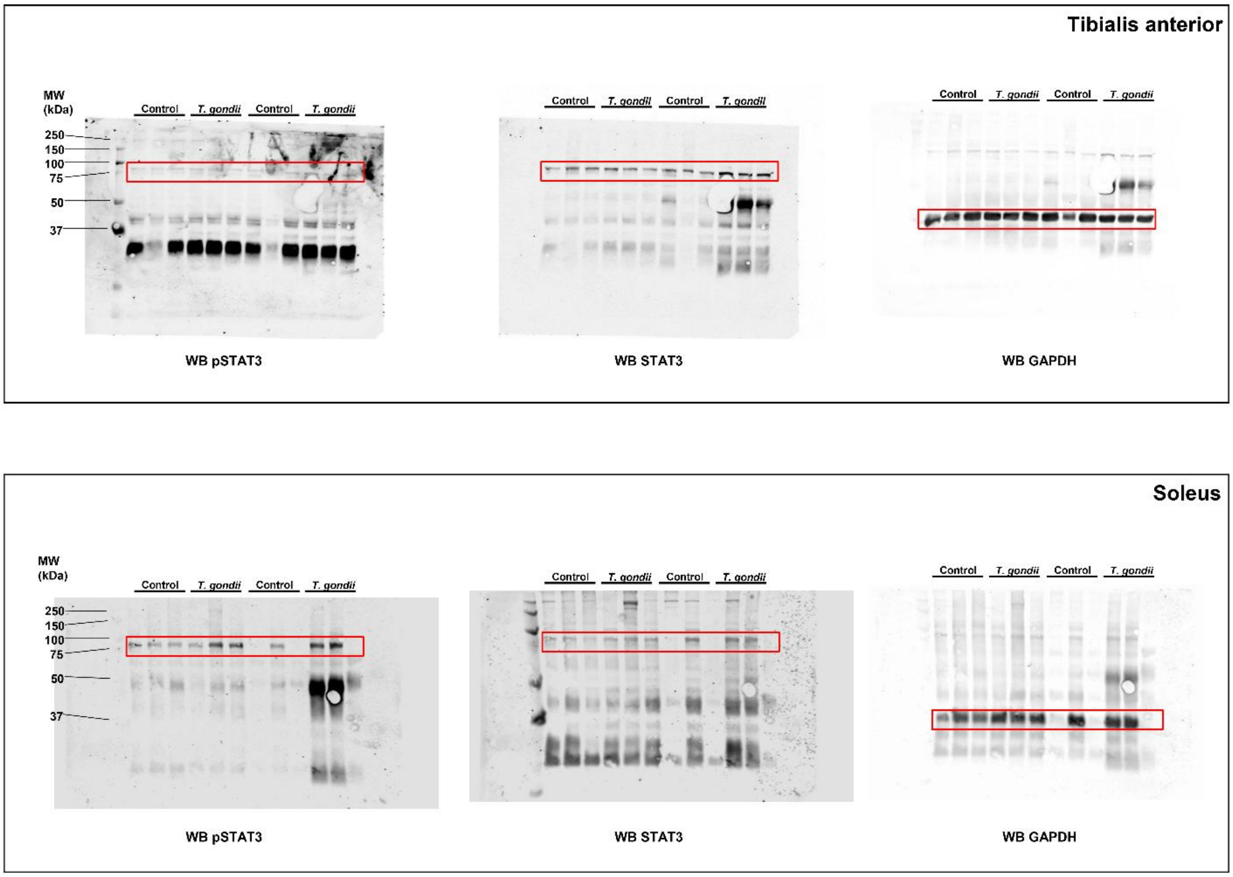

Supplementary Figure 6: Original, uncropped blots for Figure 5.

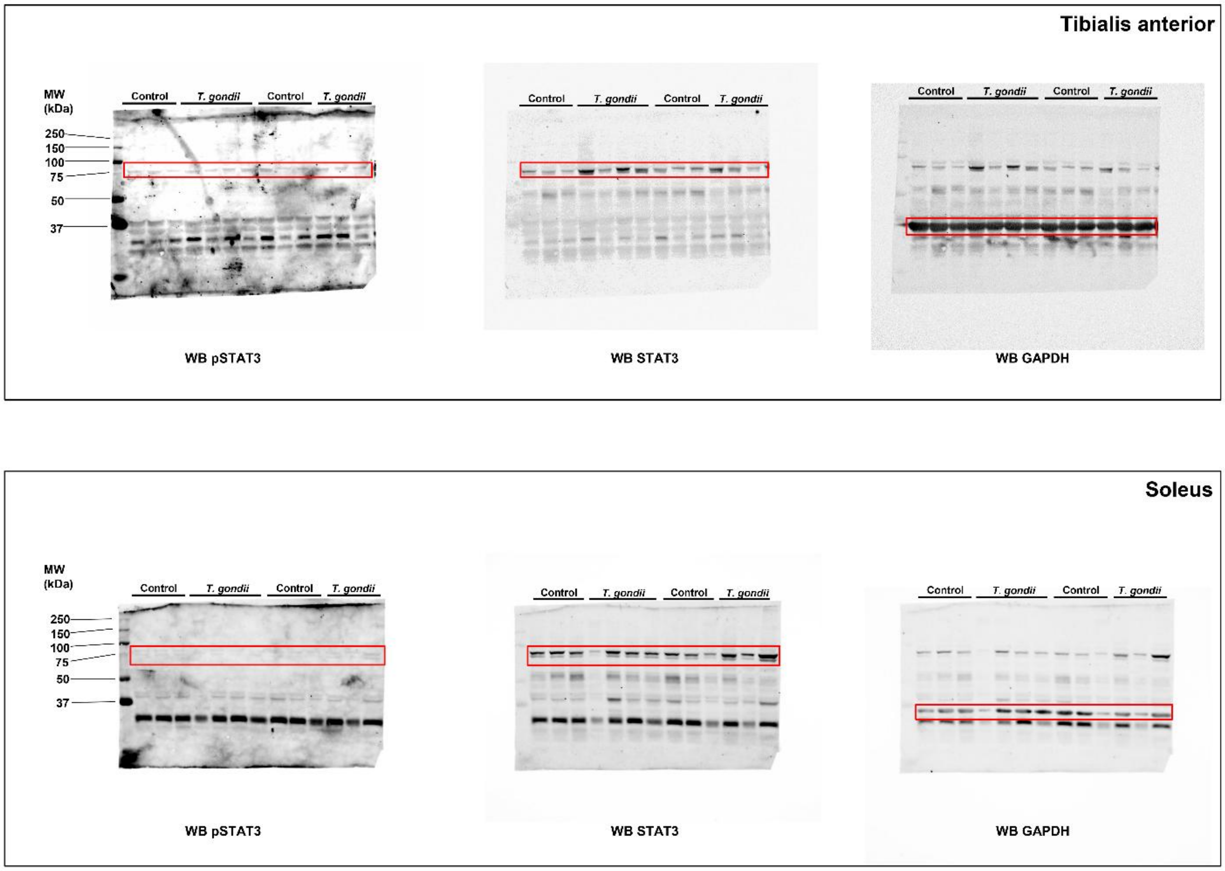

Supplementary Figure 7: Original, uncropped blots for Figure 7.
