# Supplementary material for: Physical exercise protects against Toxoplasma gondii infection-induced muscle atrophy and microvascular rarefaction
Source: Commun Biol. 2026 Mar 10;9:562. doi: 10.1038/s42003-026-09810-9 (PMC13103317; doi:10.1038/s42003-026-09810-9)
Supplement: Supplementary file 3 — Gating strategy for CBA analysis [file 42003_2026_9810_MOESM3_ESM.docx]

The CBA kit uses microbeads conjugated to specific capture antibodies for each target analyte. Each analyte is associated with a distinct bead population, characterized by a unique fluorescence intensity for the same fluorochrome (APC). This allows the different analytes in the sample to be clearly distinguished. After the beads are mixed with the sample—containing the unknown analytes—PE-labeled detection antibodies are added. These PE-conjugated antibodies bind to the analytes captured on the beads, forming a “sandwich” complex: bead – analyte – PE detection antibody.

During flow cytometry acquisition, two parameters are measured for each bead event:

1. Bead identity (based on APC fluorescence), which allows us to determine the analyte associated with that bead.

2. PE fluorescence intensity (from the detection antibody), which is proportional to the amount of analyte bound.

The kit includes lyophilized recombinant standards that are reconstituted and serially diluted to generate a standard curve. After data acquisition, dedicated software (FCAP Array) is used to analyze the results by gating bead populations, calculating MFI values, generating standard curves, and determining analyte concentrations.

The gating strategy for BD Biosciences CBA assays begins with identifying the bead population based on size and granularity using an FSC vs. SSC plot, which enables exclusion of debris and non-specific events, as illustrated in the figure below.


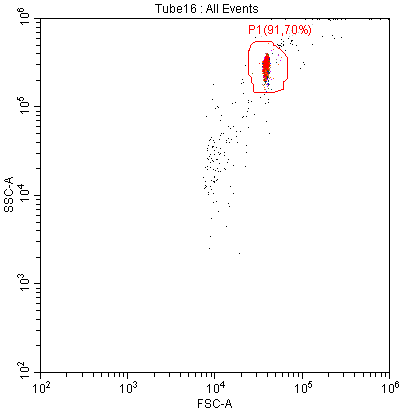


Once the bead population is isolated, other gates second are applied to the classification channel (typically APC or a far-red fluorescence detector), where each bead set forms a distinct cluster according to its characteristic fluorescence intensity, as shown in the figure below for the anti-mouse Th1/Th2/Th17 kit.


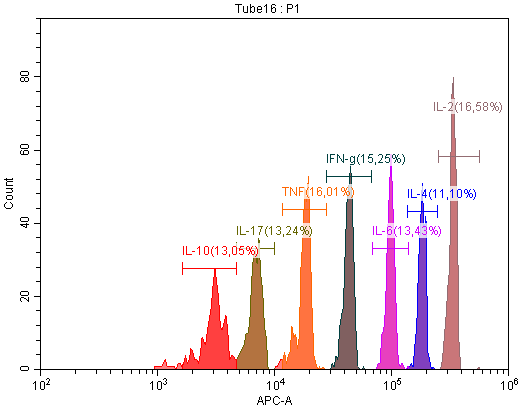


These clusters represent the individual analytes included in the assay. After the bead subsets are separated, PE fluorescence is evaluated within each gated bead population, as shown in the figure below.


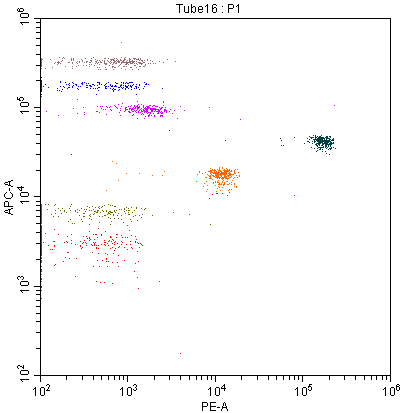


IL-2

IL-4

IL-6

IFN-γ

TNF

IL-17

IL-10

The PE signal corresponds to the amount of detection antibody bound and thus reflects the concentration of each analyte in the sample. This sequential gating approach—first isolating beads, then resolving bead subsets, and finally quantifying PE signal—ensures accurate and reliable identification and measurement of all analytes in a multiplexed CBA assay.
