# Supplementary material for: Physical exercise protects against Toxoplasma gondii infection-induced muscle atrophy and microvascular rarefaction
Source: Commun Biol. 2026 Mar 10;9:562. doi: 10.1038/s42003-026-09810-9 (PMC13103317; doi:10.1038/s42003-026-09810-9)
Supplement: Supplementary file 4 — Description of Additional Supplementary Files [file 42003_2026_9810_MOESM4_ESM.docx]

Description of Additional Supplementary Files

**File name:** Supplementary Data

**Description:** Spreadsheet with numerical values used to generate the graphs in this manuscript.
